# On the formation of current ripples

**DOI:** 10.1038/srep11390

**Published:** 2015-06-12

**Authors:** J. Bartholdy, V. B. Ernstsen, B. W. Flemming, C. Winter, A. Bartholomä, A. Kroon

**Affiliations:** 1 Department of Geosciences and Natural Resource Management, University of Copenhagen, Øster Voldgade 10, DK-1305 Copenhagen, Denmark; 2 Marine Science Department, Senckenberg Institute, Suedstrand 40, 26382 Wilhelmshaven, Germany; 3 MARUM, University of Bremen, Leobener Strasse, 28359 Bremen, Germany

## Abstract

For grain sizes finer than coarse sand, the first flow-transverse bedforms to develop are current ripples. Although numerous studies have analysed different aspects of bedform morphodynamics, to date no comprehensive physical explanation for the formation of ripples has been given. We offer such an explanation based on a virtual boundary layer concept, and present a model predicting ripple height on the basis of grain size, current velocity and water depth. The model contradicts the conventional view of current ripples as bedforms not scaling with flow depth. Furthermore, it confirms the dependence of ripple dimensions on grain size, and their relative insensitivity to flow strength.

When water flowing over sand exceeds the critical shear stress for motion, bedforms develop as a result of dynamic processes acting across the interface between sand and water. For grain sizes finer than coarse sand, the first flow-transverse bedforms to develop are current ripples. These are defined^1^ as bedforms having wavelengths smaller than 0.6 m and as not interacting with the water surface. Although numerous studies have analyzed different aspects of bedform morphodynamics since the classical pioneering work of Gilbert^2^, the current state of knowledge is even today adequately described by the statement of Costello and Southard^3^ as being “… a reasonable way of compressing our ignorance into a smaller space”. In the study of ripple formation, the wavelength of small embryonic flow-transverse “wavelets” was found to be primarily dependent on grain size^4^. The transformation into mature ripples has, amongst others, been studied by^5^,^6^,^7^,^8^,^9^. Nevertheless, to date no comprehensive physical explanation for the formation of ripples has been given. Such an explanation, however, is essential for our understanding of one of the most characteristic features associated with sediment transport and, hence, of how water is able to shape the surface of large parts of the Earth (Fig. 1) as well as other planets. We present such an explanation based on a virtual boundary layer concept.

A virtual boundary layer above dunes was described^10^, as being proportional in thickness (D′) to the bedform height, and in which the mean velocity above the mean bed level (V′) accelerates towards the bedform crest where – together with skin roughness – it controls the friction velocity acting on the crest (uf_crest_, Fig. 2):


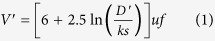



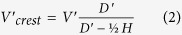



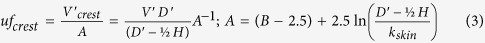


Here, H is the bedform height, ks the overall hydraulic roughness, k_skin_ = 2.5 d the skin roughness with d as the grain size^11^, and B a factor relating to the logarithmic velocity profile with B = 8.5 for rough conditions and 2.5 ln(R_*_) + 5.5 for smooth conditions where R_*_ is the skin friction Reynolds number (uf_crest_ d/ν; ν being the kinematic viscosity). The average flow over a rippled bed is generally accepted to be rough (Equation 1), whereas most flow conditions at ripple crests fall into the transition zone between rough and smooth flow (1.6 < R^*^ < 70). According to^10^, B can be estimated by means of Eq. 4 for the transition zone:


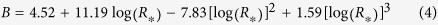


The concept of a virtual boundary layer as described above has previously been used to formulate a bed load equation based on dune migration^12^ in which the calibration constant can be explained by known physical properties.

When applying the virtual boundary layer concept to ripples, a characteristic pattern in the variation of uf_crest_ with bedform height is observed for these bed features as they grow from an infinitesimally small perturbation (wavelet) to their equilibrium size (Fig. 3). The friction velocity at the crest initially decreases from a relatively high value towards a minimum from where it then progressively increases with increasing bedform height. The reason for this is that the relative friction at the crest (k_skin_/D′_crest_) is large when the bedform height (and thus D′_crest_) is small. This causes the friction velocity at the crest to be relatively large. As the bedform grows, the relative friction at the crest decreases, and because of the logarithmic factor in Equation 3 this causes the variation of A with bedform height to take on a more curved shape than the variation of V′_crest_. As a consequence, the relation between the two parameters (and hence uf_crest_) initially strives towards a minimum value at the onset of bedform growth. Because sediment transport is directly related to a positive power of the friction velocity, ripple growth on either side of this minimum will either be enhanced with increasing bedform height (on the smaller side due to a decreasing uf_crest_ and, other things being equal, resulting deposition) or reversed (on the larger side due to an increasing uf_crest_ and, other things being equal, resulting erosion). The minimum therefore represents a dynamic equilibrium towards which small perturbations will grow before eventually reaching a mature stable bedform height.

In order to calculate V′, it is necessary to estimate the hydraulic form roughness, k_form_. This parameter occurs in the logarithmic term of Equation 1 as k_s_ = k_form_+k_skin_ and, because of that, the results are not particularly sensitive to its variation. A plot of flume data suggests the following relation between β = k_form_/H and L (Fig. 4a):


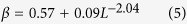



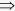



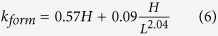


For the purpose of this study a large flume data set^13^ (8-foot-wide flume) was used. It represents the most comprehensive experimental data set on the study of bedforms ever carried out. The data were supplemented by those of^14^,^15^, except for the roughness calibration because the water slope was not recorded here. The roughness is calculated from uf = (D I g)^½^ and V on the basis of the logarithmic velocity profile with D as the water depth, I the water slope, V the mean velocity in the flume, and g the acceleration due to gravity. The resulting regression between β and ripple length (Fig. 4a) correlates with a coefficient of determination of R^2^ = 0.99.

More suitable data on small-scale ripples are needed in order to address the rapid increase of β with decreasing ripple length in this size range. The smallest ripple length in the empirical data by means of which equation 5 was calibrated is 0.12 m. This means that this length should be regarded as the smallest reliable ripple length to which the presented model can be applied. The steep increase of β with decreasing L in this size range also means a rapid increase in the uncertainty of the calculation of β and thus of k_form_. A decrease of L from 0.30 m over 0.20 m to 0.10 m causes a variation in L of +/−10% to produce a similar error range in β, which increases from +/−13% over +/−15% to +/−20%. Another feature of interest revealed by Eq. 5 and Fig. 4a is that the asymptotic nature of the relation between β and L levels out at more or less the exact wavelength range regarded as discriminating ripples from dunes^1^: 0.6–1.0 m. It is beyond the scope of this paper to go into further details here, but it is nevertheless notable that this transition coincides with a change in the relation between hydraulic roughness and bedform wavelength.

The thickness of the virtual boundary layer D′ and its relation to ripple height H was found by calibrating D′/H for the same ripple runs as well as those from^14^,^15^, concentrating on ripples only. Because of a clear tendency of D′/H getting smaller the shallower the water gets, only runs with a dimensionless flow depth (D/d) above 1000 were used. This number was chosen in order to avoid scale effects, as already suggested by^16^. The value of D′/H was then varied until the minimum value of uf_crest_ (from Eq. 3) coincided with the measured ripple height; D′/H was found to correlate with L as (Fig. 4b):


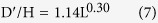


A model was developed on the basis of the above algorithms. H in the model is determined as the ripple height corresponding to the minimum value of uf_crest_ when H is increased in increments of 0.1 mm above an initial value of 5 mm.

A marked property of ripples is that their dimensions are proportional to grain size, e.g.^14^,^15^. An obvious validation procedure of the proposed model is therefore to test how well it reproduces this relation. As shown in Fig. 5a, the model is actually able to predict increasing ripple heights with increasing grain size. The reason for this is that the relative roughness in the boundary layer above the crest increases as the grain size increases. This results in a lowering of the A-curve (Fig. 3) and thereby causes the minimum value of uf_crest_ to shift towards a larger bedform.

Another well documented property of ripples is their insensitivity to changes in current velocity^14^,^15^. This is confirmed by the model prediction where ripple heights vary by less than +/−4% at current velocities ranging from 0.4 m/s to 0.6 m/s (Fig. 5b).

The conventional view about current ripples is that they apparently do not scale with flow depth. Flume studies as well as, for example, a recently published model^17^ suggest the maximum height of current ripples to be about 3 cm. This is contradicted by the fact that ripples in nature have actually been documented to reach heights of up to about 10 cm^18^. The above model provides an explanation for this. To obtain the full picture, it is necessary to also estimate realistic values of the bedform length. As suggested by Yalin^16^, ripple length is approximately equal to 1000 d. Using this approximation together with the empirical relations between L and H describing mean and maximum heights respectively^18^,^19^, the area bordered by the two equations and their gradient correspond well with the model for water depths varying between 0. 5 m and 4.0 m (Fig. 5c):


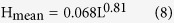



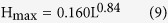


As the model relies on the existence of a logarithmic velocity profile, a maximum depth of approximately 4 m seems to be a reasonable upper boundary value. The depth-dependence follows from the fact that uf increases when flow depth decreases, other things being equal. This changes the shape of V′_crest_ as a function of H (Fig. 3) towards a steeper relation, and forces the minimum value of uf_crest_ = V_crest_/A towards lower values of H.

Unfortunately, comprehensive data sets dealing with morphodynamics of ripples in nature are scarce. Our knowledge is mostly based on flume data where variations in water depth are inherently limited. However, the data used for the construction of Figs 6 and 7 is from a natural environment. It was derived from the unpublished master thesis of one of the authors^20^ and is here presented as an original dataset. The data on H and L were collected in a small alluvial river in Denmark (Gels Å) by means of an echo-sounder mounted on a 6 m long fixed and floating frame in a mobile wagon-box connected to a data-logger which also controlled the movements of the box. The wagon-box was pulled back and forth across the 6 m long observation frame over eight 80-hour-long periods (+40 hours) by means of an electrical motor. Current velocity and temperature were measured by two self-recording current meters of the type Aanderaa RCM9. The water temperature varied between 10 and 15 °C and the bed sediment mean grain size was 0.418 mm (equivalent fall diameter).

The water depth, mean water velocity and mean ripple dimensions for each run in the course of 690 hours is presented in Fig. 6. The time series show how a drop in water depth from about 0.8 m to 0.7 m and a corresponding drop in mean velocity from between 0.5 and 0.6 m s^−1^ to between 0.4 and 0.5 m s^−1^ after 130–160 hours of observation caused a change in bed configuration from about 1.0–1.4 m long dunes to ripples fluctuating in length between 0.2 and 0.6 m. In Fig. 7 the bedform height fluctuations over each of the eight 80-hour-long recording periods have been averaged. The result shows that the model is able to predict ripple height within very small error margins. The measured mean height over the last four recording periods is 0.0318 m, whereas the corresponding model result is 0.0320 m. Although this single value cannot be regarded as a satisfactory empirical test of the model, it is nevertheless a valid indicator of its reliability. The fact that the model confirms the dependence of ripple size on grain size, in accordance with empirically derived relations^18^,^19^, and also confirms the well-known insensitivity to flow strength, supports its validity. This leads us to conclude that current ripples are generated and dimensionally scaled as a relatively simple consequence of the virtual boundary layer in association with interactions between well-known properties of the logarithmic velocity profile, grain-size and flow depth. On the other hand, the model is not able to detect the often cited upper grain-size limit for ripple formation at 0.6–0.7 mm^16^. It should be emphasized, however, that the nature of this limit is still poorly understood.

## Additional Information

**How to cite this article**: Bartholdy, J. *et al*. On the formation of current ripples. *Sci. Rep*. **5**, 11390; doi: 10.1038/srep11390 (2015).

## Figures and Tables

**Figure 1:**
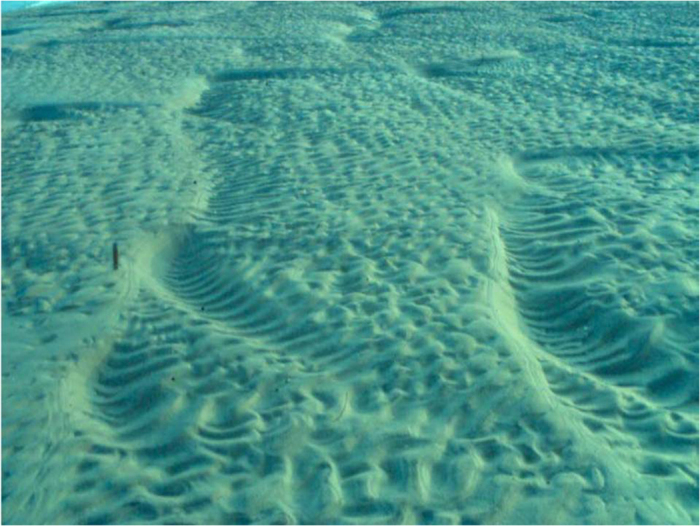
Ripples are the most common bedform type on the Earth’s surface. They occur on beaches, in rivers, on the seabed, in tidal intets and - as shown here - on a tidal flat of the Danish Wadden Sea where ripples are the dominating feature superimposed on small dunes. The rod on the left is 15 cm long. Photo, Jesper Bartholdy.

**Figure 2:**
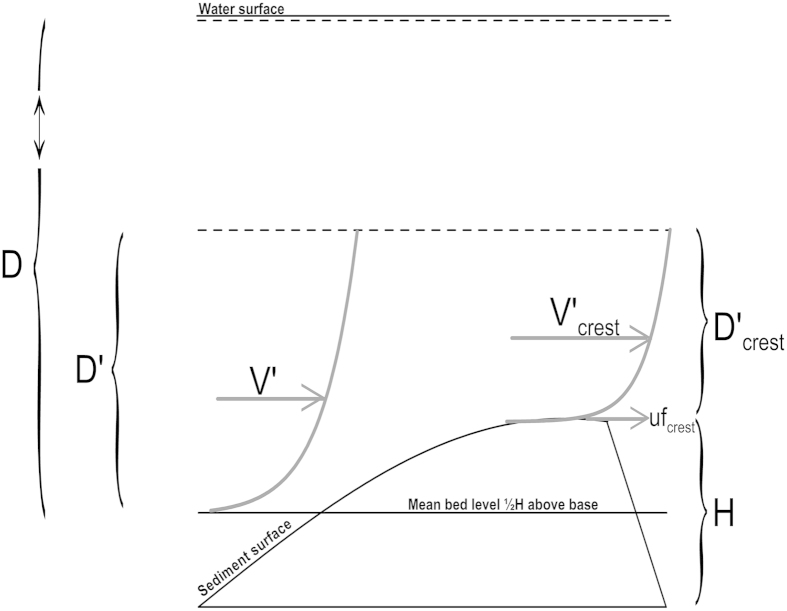
The concept of a virtual boundary layer above bedforms. The thickness of the layer D′ is smaller than or equal to the flow depth D. The mean velocity V′ within the layer increases as it contracts up the stoss side of the bedform to V′_crest_ controlling the friction velocity uf_crest_ at the bedform crest.

**Figure 3:**
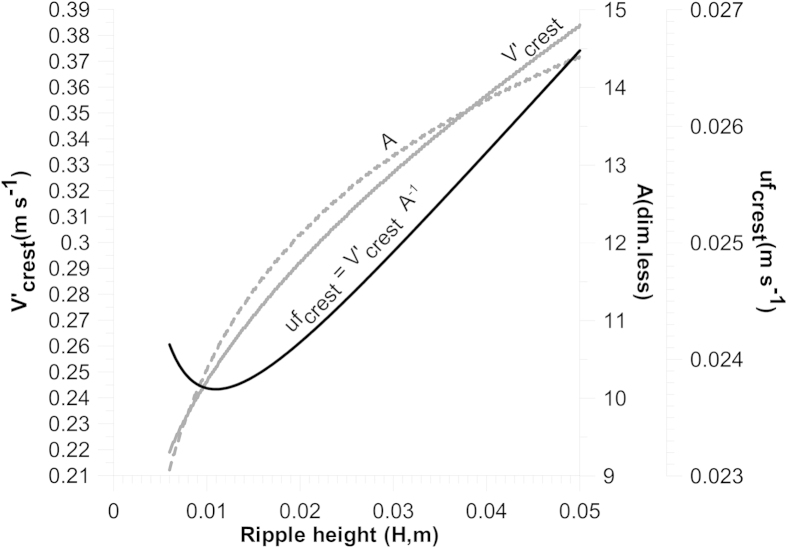
Model prediction (see text) of the terms in Equation (3) as a function of ripple height. The results are derived on the basis of data from^13^, corresponding to run 31 with 0.19 mm sand in an 8-foot-wide flume (L = 0.18 m and H = 0.012 m). Full gray line: mean velocity in the virtual boundary layer above the crest (V′_crest_); dashed gray line: A; and full black line: uf_crest_. Flow properties and grain size are kept constant, whereas the ripple height increases from values below the measured size to above it.

**Figure 4:**
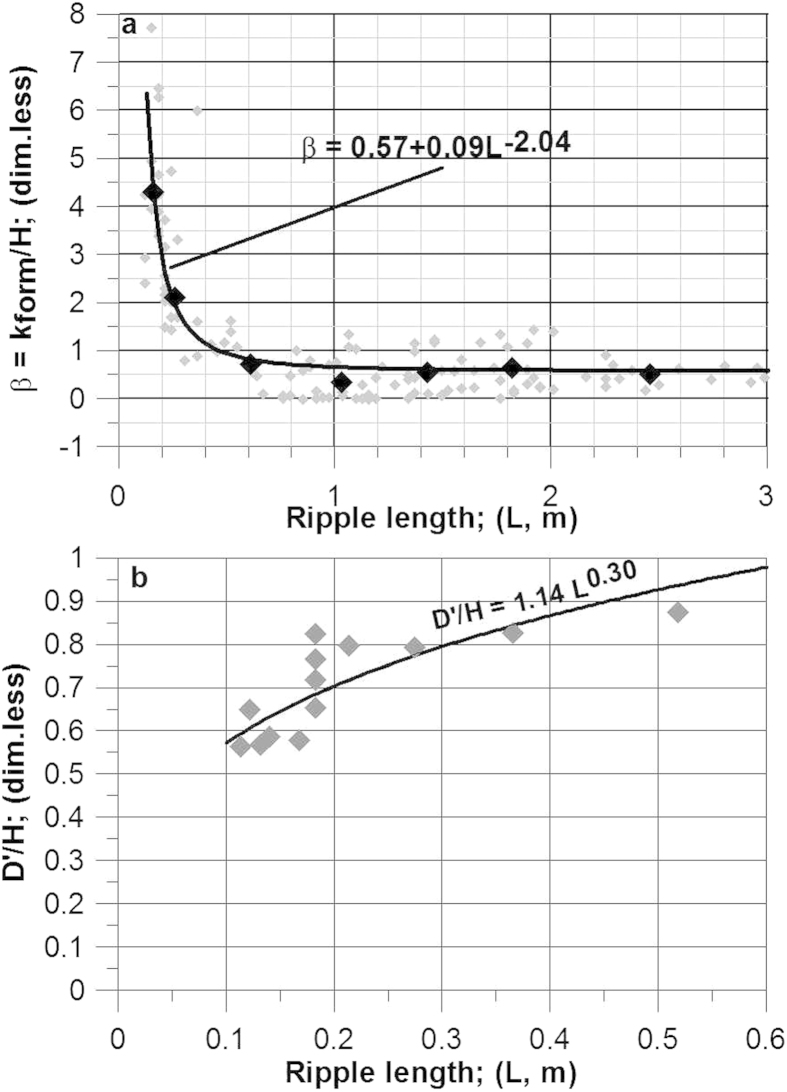
Calibration results forming the basis of the presented model. **a**) Relation between bedform length and β (see text). The data are from^13^ (8-foot-wide flume with L < 3 m). The regression relates to mean values (black symbols) calculated for the following intervals (in m): L = 0.1–0.2; 0.2–0.4; 0.4–0.8; 0.8–1.2; 1.2–1.6; 1.6–2.0; 2.0–3.0. The regression correlates with a coefficient of determination of R^2^ = 0.99.The small gray symbols represent the whole data set for L < 3 m. **b**) Values of D′/H plotted against ripple length. The regression correlates with a coefficient of determination of R^2^ = 0.65.The datasets are from^13^,^14^,^15^. Only datasets with D/d > 1000 were used. The data sets from^14^,^15^ were reduced to represent the smallest and largest current velocity at which ripples were present in the flume.

**Figure 5:**
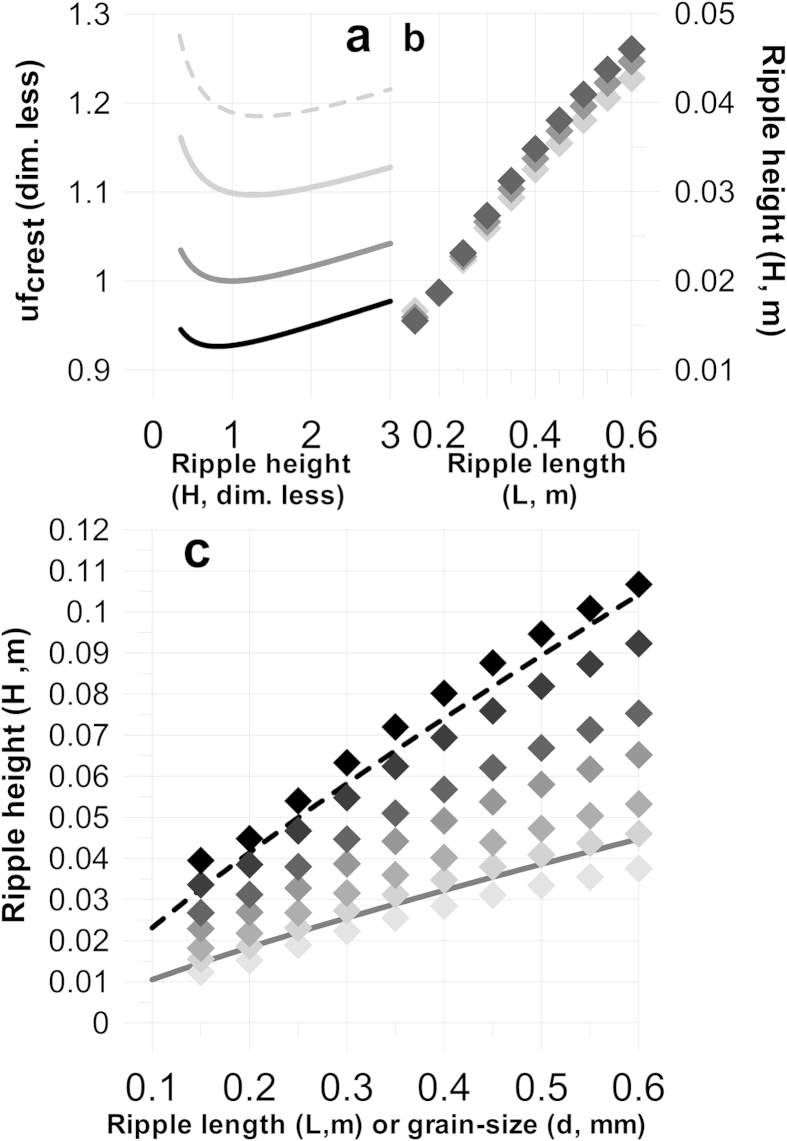
Model prediction of ripple height (H) as a function of grain-size (d), mean current velocity (V), water depth (D) and ripple length (L). **a**) Model prediction of the variation of uf_crest_ with H based on data of^13^ (Run 52, 0.27 mm sand in 8-foot-wide flume). The dark gray line represents the actual run, whereas the black (0.2 mm), light gray (0.4 mm) and dashed light gray (0.5 mm) lines represent the same data except for the grain size. All H as well as uf_crest_ values are normalized with values corresponding to the minimum of uf_crest_ in the actual run. **b**) H as a function of L = 1000 d. The light gray, dark gray and black diamond symbols represent model results with water depth D = 0.75 m and current velocity V = 0.4 m/s, 0.5 m/s and 0.6 m/s respectively. **c**) H as function of L = 1000 d compared with the algorithms suggested by^18^,^19^. The full and stippled lines represent Eq. 8 and 9 respectively. From light gray to black diamond symbols, the water depth D is varied from 0.5 m, to 0.75 m, 1.90 m, 1.5 m, 2 m, 3 m, and 4 m. V is kept constant at a value of 0.6 m/s.

**Figure 6:**
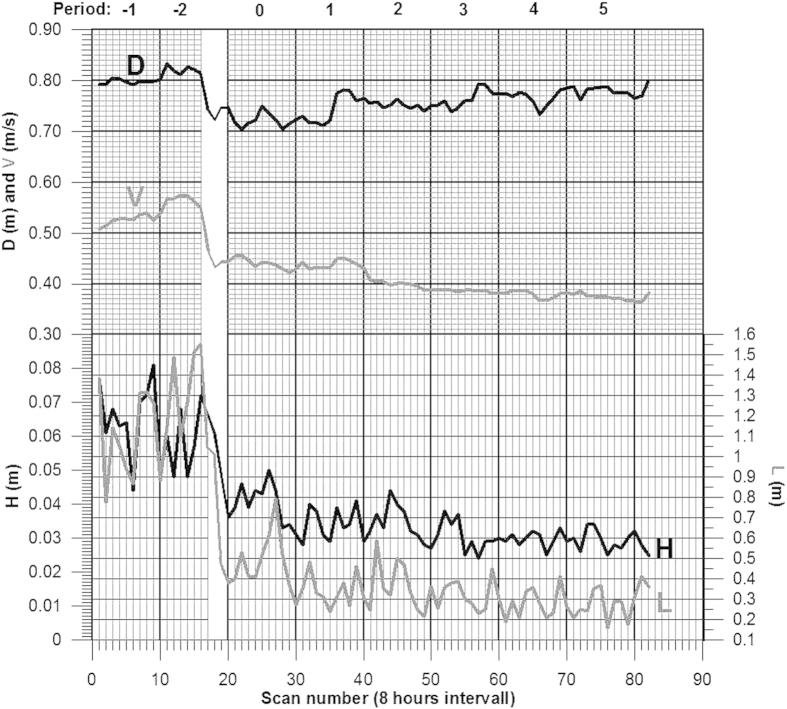
An example of ripple morphodynamics in a small alluvial river. Bedform height (H), length (L), and dynamics in form of water depth (D) and mean current velocity (V) in the river Gels Å, Denmark, during spring 2001. The time series consists of eight continuous 80-hour recording intervals over a total period of 690 hours. The blanked period of 30 hours corresponds to a transitional period from dunes to ripples caused by a drop in river flow dynamics.

**Figure 7:**
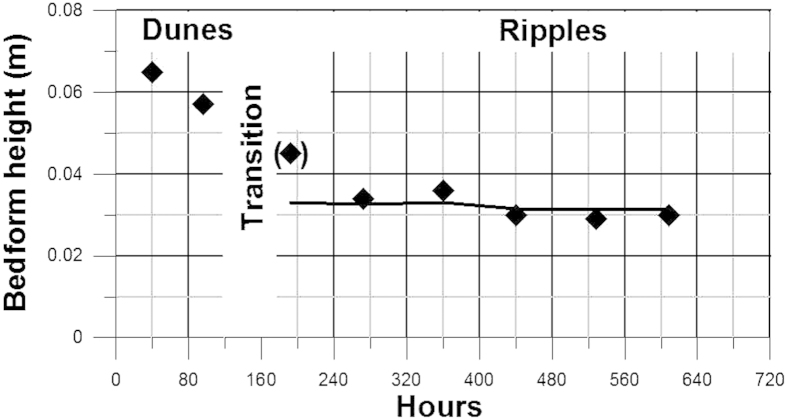
Measured mean results of bedform heights and the corresponding model results for the data presented in Fig. 6. Based on the 80-hour recording periods shown in Fig. 6, the measured mean bedform heights are indicated by diamonds and the corresponding model results by a full line.
